# Supplementary material for: Effects of Efficient Expression of Vitreoscilla Hemoglobin on Production, Monosaccharide Composition, and Antioxidant Activity of Exopolysaccharides in Ganoderma lucidum
Source: Microorganisms. 2021 Jul 21;9(8):1551. doi: 10.3390/microorganisms9081551 (PMC8401987; doi:10.3390/microorganisms9081551)
Supplement: Supplementary file 1 [file microorganisms-09-01551-s001.zip › microorganisms-1307472-Supplementary.pdf]

Supplementary information

The synthesized *Nhe*I-intron-opvhb-*Sma*I sequence

GCTAGCATGCCCGTGAGTCCTGCATCCCCATCGTGCACCGTATTCACCTCAT
CGTTTGGCCCCCTTCTCACAGGTCAAGGTCATGCTCGACCAGCAGACCATC
AACATCATCAAGGCCACCGTCCCCGTCCTCAAGGAGCACGGCGTCACCATC
ACCACCACCTTCTACAAGAACCTCTTCGCCAAGCACCCCGAGGTCCGCCCC
CTCTTCGACATGGGCCGCCAGGAGTCCCTCGAGCAGCCCAAGGCCCTCGC
CATGACGGTCCTCGCGGCCGCCCAGAACATCGAGAACCTCCCCGCCATCCT
CCCCGCCGTCAAGAAGATCGCCGTCAAGCACTGCCAGGCCGGCGTCGCCG
CCGCCCACTACCCCATCGTCGGCCAGGAGCTCCTCGGCGCCATCAAGGAG
GTCCTCGGCGACGCCGCCACCGACGACATCCTCGACGCCTGGGGCAAGGC
CTACGGCGTCATCGCCGACGTCTTTATCCAGGTCGAGGCCGACCTCTACGC
CCAGGCCGTCGAGTGACCCGGG

The *gpd* intron sequence was highlighted in red-color font

Sequence alignment of the original and optimized VHb gene.

Sequence alignment

5' Flanking

```
Protein        M  L  D  Q  Q  T  I  N  I  I  K  A  T  V  P  V  L  K  E  H  G  V  T  I  T  T  T  F  Y  K
Original 1     ATGTTAGACCAGCAAACCATTAACATCATCAAAGCCACTGTTCCTGTATTGAAGGAGCATGGCGTTACCATTACCACGACTTTTTATAAA
Optimized      ATGCTCGACCAGCAGACCATCAACATCATCAAGGCCACCGTCCCCGTCCTCAAGGAGCACGGCGTCACCATCACCACCACCTTCTACAAG

Protein        N  L  F  A  K  H  P  E  V  R  P  L  F  D  M  G  R  Q  E  S  L  E  Q  P  K  A  L  A  M  T
Original 91    AACTTGTTTGCCAAACACCCTGAAGTACGTCCTTTGTTTGATATGGGTCGCCAAGAATCTTTGGAGCAGCCTAAGGCTTTGGCGATGACG
Optimized      AACCTCTTCGCCAAGCACCCCGAGGTCCGCCCCCTCTTCGACATGGGCCGCCAGGAGTCCCTCGAGCAGCCCAAGGCCCTCGCCATGACG

Protein        V  L  A  A  A  Q  N  I  E  N  L  P  A  I  L  P  A  V  K  K  I  A  V  K  H  C  Q  A  G  V
Original 181   GTATTGGCGGCAGCGCAAAACATTGAAAATTTGCCAGCTATTTTGCCTGCGGTCAAAAAAATTGCAGTCAAACATTGTCAAGCAGGCGTG
Optimized      GTCCTCGCGGCCGCCCAGAACATCGAGAACCTCCCCGCCATCCTCCCCGCCGTCAAGAAGATCGCCGTCAAGCACTGCCAGGCCGGCGTC

Protein        A  A  A  H  Y  P  I  V  G  Q  E  L  L  G  A  I  K  E  V  L  G  D  A  A  T  D  D  I  L  D
Original 271   GCAGCAGCGCATTATCCGATTGTCGGTCAAGAATTGTTGGGTGCGATTAAAGAAGTATTGGGCGATGCCGCAACCGATGACATTTTGGAC
Optimized      GCCGCCGCCCACTACCCCATCGTCGGCCAGGAGCTCCTCGGCGCCATCAAGGAGGTCCTCGGCGACGCCGCCACCGACGACATCCTCGAC

Protein        A  W  G  K  A  Y  G  V  I  A  D  V  F  I  Q  V  E  A  D  L  Y  A  Q  A  V  E  *
Original 361   GCGTGGGGCAAGGCTTATGGCGTGATTGCAGATGTGTTTATTCAAGTGGAAGCAGATTTGTACGCTCAAGCGGTTGAATAA
Optimized      GCCTGGGGCAAGGCCTACGGCGTCATCGCCGACGTCTTTATCCAGGTCGAGGCCGACCTCTACGCCCAGGCCGTCGAGTGA
```

3' Flanking
